# Gender Differences in the Association between Workplace Bullying and Depression among Korean Employees

**DOI:** 10.3390/brainsci13101486

**Published:** 2023-10-20

**Authors:** Sra Jung, Hee-Jun Lee, Mi Yeon Lee, Eun Soo Kim, Sang-Won Jeon, Dong-Won Shin, Young-Chul Shin, Kang-Seob Oh, Min-Kyoung Kim, Sung Joon Cho

**Affiliations:** 1 Department of Psychiatry, Cha University Ilsan Medical Center, Goyang 10223, Republic of Korea; srajung890@chamc.co.kr; 2 Department of Psychiatry, Medical Corps of the 6th Marine Brigade, Republic of Korea Navy, Incheon 23103, Republic of Korea; 21heedong@gmail.com; 3 Department of Biostatistics, Kangbuk Samsung Hospital, Sungkyunkwan University School of Medicine, Seoul 03181, Republic of Korea; 4 Department of Psychiatry, Kangbuk Samsung Hospital, Sungkyunkwan University School of Medicine, Seoul 03181, Republic of Korea; 5 Workplace Mental Health Institute, Kangbuk Samsung Hospital, Seoul 03181, Republic of Korea

**Keywords:** workplace mental health, workplace bullying, depression, gender difference

## Abstract

Workplace bullying is a prevalent issue with a significant impact on employees’ mental health. This study aimed to explore the relationship between workplace bullying and the prevalence of depression, with a particular focus on the role of gender. A total of 12,344 Korean employees aged 19–65 years were included in the study. They completed the Center for Epidemiologic Studies Depression Scale (CES-D) and a score of 16 or higher in CES-D indicated depression. The association between workplace bullying and depression was analyzed using logistic regression analyses. The average CES-D scores were higher for both male and female employees who experienced bullying than for those who did not (*p* < 0.001). The association between the experience of workplace bullying and the prevalence of depression was statistically significant for both genders, with a stronger correlation observed among male employees (*p* for interaction < 0.001). Organizations are urged to address workplace bullying, particularly for male employees, through the implementation of anti-bullying strategies and policies, as well as the provision of mental health resources and support.

## 1. Introduction

Workplace bullying is a serious problem that can adversely affect the physical and mental health of employees [1]. It involves repetitive aggressive behaviors that target individuals or groups and cause physical or psychological harm. This can lead to decreased job satisfaction and work performance and increased absenteeism and turnover rates [2]. Numerous studies [3,4,5] have demonstrated the association between workplace bullying and an increased risk of depressive symptoms among employees. For example, a study conducted in Germany found that workplace bullying, particularly when perpetrated by coworkers, was associated with an increased risk of depressive symptoms among employees [6]. Similarly, a study conducted among Taiwanese nurses found that workplace bullying is positively associated with depression [7]. Unlike workplace cultures in other countries, Korean workplace culture, which emphasizes interpersonal relationships due to high levels of power distance, Confucianism, and collectivism [8], can make workplaces prone to bullying [9]. In Korea, conflict with colleagues rather than supervisors emerged as a more potent predictor of workplace bullying, indicating the unique dynamics at play [10]. Additionally, unlike in the United Kingdom, factors such as job type and part-time employment status had little influence on bullying scores [10]. Furthermore, stress from workplace bullying can spill over into the home lives of Korean employees, leading to greater work-to-family conflict [9]. These unique cultural and social contexts can make the impact of workplace bullying particularly profound in Korean society. These peculiarities highlight the need for further research to better understand the unique characteristics of workplace bullying in Korean contexts, which are not yet well explored.

The prevalence of depression is higher in female employees than in male employees [11], and research suggests that this difference is influenced not only by sex hormonal variations but also by social and environmental factors, such as pregnancy and childbirth [12]. Additionally, there are gender differences in patterns of workplace bullying [13]. Studies indicate that male employees are more likely to report experiencing physical bullying, while female employees report emotional and relationship-related workplace bullying more frequently [14]. Moreover, research suggests that male employees tend to underreport workplace bullying compared to female employees [15]. These differences may be particularly pronounced in South Korea, influenced by the enduring impact of Confucian culture, which has historically emphasized clear gender roles [16,17]. In this cultural context, men have been primarily responsible for economic work throughout their careers, while women have been expected to manage household responsibilities [18]. Although these roles have become less rigid in modern society, the influence of Confucian values persists, contributing to a gender-biased workplace culture. In workplaces that are often male-dominated, female employees frequently experience inequality, face obstacles to career advancement, and are more likely to leave their jobs after marriage [19]. Furthermore, South Korea has grappled with workplace bullying issues known as “Taeum”, a culture rooted in Confucianism, where superiors often subject their subordinates, particularly female nurses, to inhumane treatment, such as assigning impossible tasks and offering no guidance [20,21]. This highlights the need for a separate examination of the relationship between workplace bullying and depression among male and female employees in South Korean society. However, it is worth noting that there is a shortage of research specifically exploring gender differences in the relationship between workplace bullying and depression in South Korean society.

Existing research frequently overlooks the influence of gender differences in workplace bullying. When gender differences are considered, the validity of findings related to gender-specific experiences of workplace bullying can be questioned because of the relatively small number of subjects analyzed separately by gender or significant discrepancies in the number of male and female participants [22]. For a more comprehensive understanding of the relationship between workplace bullying and the prevalence of depression among Korean employees, this study included a large dataset of Korean employees from various industries. In addition, this study explored the gender differences in this relationship, which have been identified as an important factor in previous research [23]. However, there is still an ongoing debate about the role of gender in the relationship between workplace bullying and depression. Therefore, we propose the following hypotheses:

**H1.**  *Workplace bullying is positively associated with the prevalence of depression among Korean employees*.

**H2.**  *The relationship between workplace bullying and the prevalence of depression is moderated by gender*.

## 2. Materials and Methods

### 2.1. Participants

**Figure 1:**
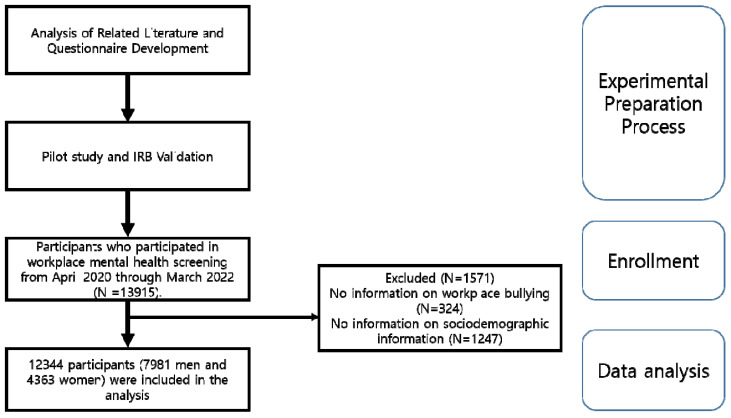
Flowchart of the recruitment process.

Our study initially included a diverse group of 13,915 employees, both male and female, aged 19 to 65 years, who participated in workplace mental health screenings at the Workplace Mental Health Institute of Kangbuk Samsung Hospital, Seoul, Republic of Korea, from April 2020 through March 2022. After excluding 1571 participants due to incomplete information on workplace bullying or sociodemographic data, the final analysis was based on a total of 12,344 participants, comprising 7981 men and 4363 women. These participants were employees of one of the country’s 18 public institutions or large corporations and voluntarily agreed to participate in mental health examinations at the request of their respective companies. The detailed participant selection process is illustrated in Figure 1.

All procedures related to the study received approval from the Institutional Review Board of Kangbuk Samsung Hospital. The study was conducted in strict accordance with the most recent version of the Declaration of Helsinki and principles of Good Clinical Practice (approval number: KBSMC 2022-03-046). The need for informed consent was waived given that the study utilized only de-identified data that were routinely collected during health screening visits.

### 2.2. Clinical Assessments

Sociodemographic factors were gathered, encompassing factors such as age, gender, level of education (college graduate or below, university graduate, master’s degree or above), and marital status (classified as married, unmarried, or other [including divorced, widowed, or separated]). We also gathered job-related demographic information, such as the duration of employment at the current workplace (in years), weekly work hours, and monthly income (categorized as below 3 million KRW, between 3 and 4 million KRW, and 4 million KRW or above).

To assess workplace bullying, we asked participants the following question: “Have you experienced intentional humiliation, harassment, or verbal abuse, or intentional social exclusion or isolation at work in the past 6 months?” Participants responded with either “Yes” or “No”. Those who responded “Yes” were defined as having experienced workplace bullying.

Depressive symptoms were assessed using the Korean version of the 20-item Center for Epidemiological Studies Depression (CES-D) scale [24,25]. This self-reported questionnaire uses a 4-point Likert scale ranging from 0 to 3 points, with higher total scores indicating more severe depressive symptoms. Cronbach’s alpha of the CES-D in this study was 0.765. Traditionally, a CES-D score of 16 or higher has been used as a threshold for depression screening [26]. Thus, in this study, we identified individuals with a CES-D score of 16 or higher as having depression.

### 2.3. Statistical Analysis

The baseline characteristics of the study groups were compared using independent *t*-tests for continuous variables and the χ^2^ test for categorical variables. The prevalence of depression in each group classified by the experience of workplace bullying was compared using the χ^2^ test. Adjusted means (standard error [SE]) of CES-D values between groups with and without workplace bullying experience were compared using analysis of covariance (ANCOVA) after adjusting for possible confounding factors such as age, education status, marital status, years of service, working hours, and income. Multiple logistic regression analyses were performed with adjustments for possible confounding variables to determine the association between workplace bullying experiences and depression. The interaction by gender difference was conducted using the likelihood ratio test to compare models with and without multiplicative interaction terms. The level of statistical significance was set at a two-tailed *p*-value < 0.05. All analyses were conducted using IBM SPSS version 28.0 (IBM Co., Armonk, NY, USA).

## 3. Results

### 3.1. Baseline Demographic Characteristics

In total, 12,344 participants were included, comprising 7981 (64.7%) male and 4363 (35.3%) female employees. The mean age of the male employees (37.32 ± 9.64) was significantly older than that of the female employees (35.45 ± 9.13). Male employees had significantly more years of service (t = 3.62, *p* < 0.001) and weekly working hours (t = 6.62, *p* < 0.001) than female employees. While the proportion of highly educated individuals was higher among female employees (χ^2^ = 222.80, *p* < 0.001), male employees constituted a higher percentage of those earning higher monthly salaries (χ^2^ = 474.04, *p* < 0.001). The proportion of female employees who experienced workplace bullying was higher (χ^2^ = 157.39, *p* < 0.001). The mean CES-D score of female employees was higher than that of male employees (t = −20.58, *p* < 0.001), and the prevalence of depression was higher in female employees than in male employees (χ^2^ = 550.95, *p* < 0.001) (Table 1).

The baseline characteristics of the groups with and without workplace bullying according to gender are shown in Table 2. Among male employees, those who experienced workplace bullying were significantly older (t = −5.82, *p* < 0.001), more highly educated individuals (χ^2^ = 12.31, *p* = 0.006), more likely to be married (χ^2^ = 19.88, *p* < 0.001), had more years of service (t = −2.28, *p* = 0.023) and weekly working hours (t = −3.37, *p* = 0.001), and received higher monthly salaries (χ^2^ = 9.25, *p* = 0.026) than non-bullied male employees. Among female employees who experienced workplace bullying, there were no significant differences in age, education level, or years of service compared to non-bullied female employees. However, bullied female employees were more likely to be unmarried individuals (χ^2^ = 16.37, *p* = 0.001), had more weekly working hours (t = −2.69, *p* = 0.007), and had lower monthly salaries (χ^2^ = 8.87, *p* = 0.031). Among both genders, those who experienced workplace bullying exhibited more severe depressive symptoms (male employees, t = −24.76, *p* < 0.001; female employees, t = −16.41, *p* < 0.001) and had a higher prevalence of depression (male employees, χ^2^ = 500.64, *p* < 0.001; female employees, χ^2^ = 177.69, *p* < 0.001) compared to non-bullied employees.

### 3.2. Comparison of the Prevalence of Depression and CES-D Scores between Bullied and Non-Bullied Groups

The prevalence of depression between groups with and without workplace bullying experiences is presented in Table 2. The prevalence of depression among male employees was significantly higher in the bullied group (71.1%) than in the non-bullied group (31.9%). Among female employees, the bullied group had a higher prevalence of depression (78.8%) than the non-bullied group (53.2%).

**Figure 2:**
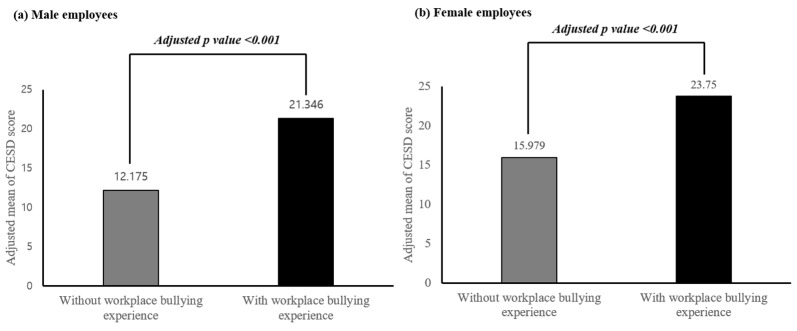
Comparison of adjusted mean CES-D scores between without and with workplace bullying experience groups in (**a**) male employees and (**b**) female employees. The adjusted means of the CES-D scores in the groups were estimated using an ANCOVA after adjusting for age, education status, marital status, years of service, working hours, and income.

Figure 2 shows a comparison of CES-D scores (as a continuous variable) according to the experience of workplace bullying among male and female employees. In male employees, after adjusting for possible confounding factors such as age, education status, marital status, years of service, working hours, and income, the adjusted mean of the CES-D was significantly higher for the bullied group (adjusted mean [SE] = 21.35 [0.31]) than for the non-bullied group (adjusted mean [SE] = 12.18 [0.11]) (adjusted *p* < 0.001), respectively. Among female employees, the bullied group (adjusted mean [SE] = 23.75 [0.39]) had a higher adjusted mean CES-D score than the non-bullied group (adjusted mean [SE] = 15.98 [0.18]) (adjusted *p* < 0.001).

### 3.3. Association between Depression and the Experience of Workplace Bullying

The results of the logistic regression analysis used to examine the factors associated with the prevalence of depression are described in Table 3 and Table 4. To explain the prevalence of depression, Model 1 included variables such as age, educational level, marital status, years of service, working hours, and monthly salary. In Model 2, the presence of workplace bullying experience was added to examine whether the explanatory power increased. For both male and female employees, the results showed an increase in the explanatory power of the models, which progressed from Model 1 (male employees, 2.0%; female employees, 5.2%) to Model 2 (male employees, 9.6%; female employees, 10.4%).

Table 5 shows the results of the multivariate logistic regression analyses after adjusting for confounding factors for the association between workplace bullying experiences and the prevalence of depression. For both male and female employees, in the fully adjusted model, bullied employees were at an increased risk of having depression than non-bullied employees (adjusted OR [95% CI] for male employees, 5.23 [4.46–6.13]; for female employees, 3.24 [2.69–3.89]). The association between workplace bullying experiences and depression was stronger for male employees than for female employees (*p* for interaction < 0.001) (Table 5).

## 4. Discussion

Our study examined the relationship between workplace bullying and the prevalence of depression among Korean employees, with a focus on understanding the role of gender in this relationship. We found a significant positive association between workplace bullying and the prevalence of depression. Notably, this association was more pronounced among male employees. These findings not only provide important insights into the gender-specific effects of workplace bullying on mental health but also mark the first large-scale examination of the differing impacts of workplace bullying on the prevalence of depression among male and female employees in the Republic of Korea.

In our study, female employees reported significantly higher instances of workplace bullying compared to their male counterparts. Male employees who had experienced workplace bullying tended to be older, more educated, married, had more years of service, had more weekly working hours, and earned a higher income. Conversely, female employees who experienced workplace bullying tended to be unmarried, had more weekly working hours, and earned lower incomes. These patterns suggest that female employees might face bullying in the early career stages, while male employees could face bullying later due to workplace competition, especially among higher-educated and higher-income individuals. This is consistent with other studies showing that regardless of the field of employment, a larger number of female employees are bullied in the workplace; moreover, female employees are more vulnerable to becoming targets of workplace bullying, especially when they work in lower hierarchical positions within organizations [27]. Conversely, male employees experience bullying regardless of their positions within their organizations [27]. This highlights the importance of ongoing efforts to understand and address the gender-specific dynamics and impacts of workplace bullying.

Our findings demonstrate that employees of both genders who have experienced workplace bullying also experience significantly more severe and prevalent depressive symptoms. Specifically, workplace bullying increased the risk of depression by 5.23 times and 3.24 times for male and female employees, respectively. This finding highlights workplace bullying as a major risk factor for depression. These results align with those of several studies conducted in Italy [1], Norway [22], Germany [28,29], Finland [30], Denmark [31,32], and Malaysia [33], which reported a significant relationship between workplace bullying and the prevalence of depression. In the Republic of Korea, research on call center employees has demonstrated a significant correlation between workplace bullying and an increased prevalence of depression [34]. This positive association observed between workplace bullying and the prevalence of depression can be explained by various stress models that highlight the negative effects of prolonged stress on both physical and mental health. Workplace bullying can be considered a source of prolonged social defeat stress that may affect emotional well-being, likely through changes in neuroendocrine, autonomic, and immune functions [32,35]. One study found that workplace bullying was indirectly related to life satisfaction via job-related anxiety and insomnia [36]. Additionally, prolonged periods of stress resulting from workplace bullying may lead to the development of mood disorders and increased allostatic load, which can negatively impact both physical and mental health [37].

Our novel finding is that workplace bullying substantially increased the prevalence of depression for both genders, with a notably higher risk for male employees than for female employees. Previous studies on the gender-specific impact of bullying on mental health have reported mixed outcomes. One study suggested that being male could serve as a protective factor, preserving mental health despite experiencing workplace bullying [23]. However, another study found that while female employees generally reported experiencing more workplace bullying [22,38], the impact on mental health was more pronounced among male employees [22], which aligned with our findings. Similarly, a study conducted among Italian employees found that although female employees were more likely to perceive bullying, the association between severe forms of bullying and mental disorders was stronger among male employees [39].

There are two primary explanations for the greater impact of workplace bullying on the mental health of male employees. First, the impact of workplace bullying on male employees’ mental health may be exacerbated by the perception that their success in the workplace is under threat. Men often associate their roles as fathers and husbands with economic contributions to the family [40]. Therefore, unemployment and workplace instability significantly affect mental health [41]. Experiencing bullying at work could be perceived as a threat to their ability to fulfill this role, potentially leading to feelings of hopelessness [38]. In our study, male employees who were more educated, worked more hours, and had higher incomes reported more instances of workplace bullying, suggesting that bullying could become more prevalent as they gained a competitive advantage over time. This perception is especially pronounced in Korea’s collectivist culture, where professional success and career progression rely heavily on interpersonal relationships at the workplace. Second, male employees may have a higher threshold for reporting workplace bullying, particularly when studies rely on self-reporting methods [42]. Consequently, it takes more exposure for a man to admit to being bullied, and the level of exposure to bullying behaviors is higher for men, with more serious consequences [42]. This could be particularly true for male Korean employees, most of whom have mandatory military experience and are influenced by patriarchal norms that equate resilience with masculinity and view admitting to being bullied as a sign of weakness. In our dataset, the proportion of male employees who reported bullying experiences was notably lower than that of female employees, which supports this explanation. Understanding these gender differences and cultural nuances is crucial for addressing workplace bullying and its impact on mental health.

Our study’s findings hold substantial implications at both individual and organizational levels, particularly underscoring the need for increased awareness and preventive measures against workplace bullying. The revealed significant relationship between workplace bullying and depression, especially among men, calls for individual vigilance to symptoms and early intervention for mental health care. At an organizational level, the results emphasize the importance of implementing effective policies, preventive measures, and educational programs to combat workplace bullying and foster a supportive work environment [43]. Especially in Korea, where all males, including those in their twenties, are obliged to serve in the military, the implications of our study’s findings become profound, given the current issues surrounding mental health and suicide rates among soldiers [44]. Moreover, theoretically, our study enhances the current literature by revealing gender as a potential moderating factor in the relationship between workplace bullying and depression, offering a novel perspective and opening avenues for future research.

This study had several limitations. First, the participants selected from among those who underwent mental health examinations at Kangbuk Samsung Hospital may not fully represent all employees, potentially affecting the generalizability of our findings. Although our study did not differentiate participants by occupation, this broad approach uniquely contributes to the understanding of employee mental health in general. Future research could benefit from exploring specific occupational groups. Second, our study’s cross-sectional design limited our ability to establish causality between workplace bullying and the prevalence of depression, suggesting the need for longitudinal studies. Despite controlling for several demographic variables, unaccounted-for and confounding variables may still have existed. Finally, our reliance on self-reported measures could have introduced response bias, although we excluded careless responses. Future investigations could enhance accuracy by using objective measures to study the effects of workplace bullying on the prevalence of depression.

## 5. Conclusions

Our study provides evidence of a significant positive association between workplace bullying and the prevalence of depression among Korean employees, and this association is more pronounced among male employees. The results highlight the need to address mental health issues, potentially through the provision of resources, support services, and the development of anti-bullying policies, given the association between workplace bullying and the prevalence of depression among male employees. Our findings have significant implications, especially for Korean males required to complete military service, where depression and suicide have been identified as potential issues [44]. Future research could expand on our work by exploring the relationship between workplace bullying and the prevalence of depression across diverse demographic groups using various methodologies. The role of other potential factors and the effectiveness of various interventions in this relationship should also be explored to develop more effective strategies for preventing workplace bullying and promoting employee well-being.

## Figures and Tables

**Table 1:** Baseline characteristics in participants.

Characteristics	Overall (*n* = 12,344)	Male (*n* = 7981)	Female (*n* = 4363)	*p* Value
Age (years), mean ± SD	36.66 ± 9.50	37.32 ± 9.64	35.45 ±9.13	<0.001
Education (graduate)				<0.001
College graduate or below, *n* (%)	2957 (24.0)	2223 (27.9)	734 (16.8)	
University graduate, *n* (%)	7381 (59.8)	4488 (56.2)	2893 (66.3)	
Master’s degree or higher, *n* (%)	1959 (15.9)	1258 (15.8)	701 (16.1)	
Not specified, *n* (%)	47 (0.4)	12 (0.2)	35 (0.8)	
Marital status				<0.001
Married, *n* (%)	6815 (55.2)	4763 (59.7)	2052 (47.0)	
Unmarried, *n* (%)	5266 (42.7)	3082 (38.6)	2184 (50.1)	
Other, *n* (%)	216 (1.7)	124 (1.6)	92 (2.1)	
Missing, *n* (%)	47 (0.4)	12 (0.2)	35 (0.8)	
Years of service (years), mean ± SD	10.50 ± 9.11	10.72 ± 9.16	10.10 ± 9.01	<0.001
Hours of work per week (hours), mean ± SD	46.77 ± 7.50	47.11 ± 7.40	46.17 ± 7.63	<0.001
Monthly earned income (million KRW)				<0.001
Less than 3 million KRW, *n* (%)	3245 (26.3)	1620 (20.3)	1625 (37.2)	
3–4 million KRW, *n* (%)	3393 (27.5)	2221 (27.8)	1172 (26.9)	
Over 4 million KRW, *n* (%)	4890 (39.6)	3586 (44.9)	1304 (29.9)	
Missing, *n* (%)	816 (6.6)	554 (6.9)	262 (6.0)	
Bullying experience				<0.001
Negative	10,689 (86.6)	7138 (89.4)	3551 (81.4)	
Positive	1655 (13.4)	843 (10.6)	812 (18.6)	
Clinical characteristics				
CES-D score, mean ± SD	14.49 ± 10.01	13.13 ± 9.40	17.36 ± 10.64	<0.001
Depression +, *n* (%)	5408 (43.8)	2878 (36.1)	2530 (58.0)	<0.001
Depression -, *n* (%)	6936 (56.2)	5103 (63.9)	1833 (42.0)	

SD, standard deviation; CES-D, Center for Epidemiological Studies Depression.

**Table 2:** Baseline characteristics according to bullying experiences for males and females.

Characteristics	Male			Female		
	Bullying − (*n* = 7138)	Bullying + (*n* = 843)	*p* Value	Bullying − (*n* = 3551)	Bullying + (*n* = 812)	*p* Value
Age (years), mean ± SD	37.12 ± 9.70	39.02 ± 8.88	<0.001	35.47 ± 9.15	35.36 ± 9.05	0.756
Education (graduate)			0.006			0.164
College graduate or below, *n* (%)	2030 (28.4)	193 (22.9)		606 (17.1)	128 (15.8)	
University graduate, *n* (%)	3988 (55.9)	500 (59.3)		2344 (66.0)	549 (67.6)	
Master’s degree or higher, *n* (%)	1110 (15.6)	148 (17.6)		577 (16.2)	124 (15.3)	
Not specified, *n* (%)	10 (0.1)	2 (0.2)		24 (0.7)	11 (1.4)	
Marital status			<0.001			0.001
Married, *n* (%)	4208 (59.0)	555 (65.8)		1715 (48.3)	337 (41.5)	
Unmarried, *n* (%)	2814 (39.4)	268 (31.8)		1743 (49.1)	441 (54.3)	
Other, *n* (%)	106 (1.5)	18 (2.1)		69 (1.9)	23 (2.8)	
Missing, *n* (%)	10 (0.1)	2 (0.2)		24 (0.7)	11 (1.4)	
Years of service (years), mean ± SD	10.64 ± 9.18	11.40 ± 8.94	0.023	10.17 ± 9.06	9.80 ± 8.77	0.295
Hours of work per week (hours), mean ± SD	47.00 ± 7.28	48.01 ± 8.35	0.001	46.00 ± 7.41	46.87 ± 8.49	0.007
Monthly earned income (million KRW)			0.026			0.031
Less than 3 million KRW, *n* (%)	1482 (20.8)	138 (16.4)		1294 (36.4)	331 (40.8)	
3–4 million KRW, *n* (%)	1972 (27.6)	249 (29.5)		952 (26.8)	220 (27.1)	
Over 4 million KRW, *n* (%)	3188 (44.7)	398 (47.2)		1094 (30.8)	210 (25.9)	
Missing, *n* (%)	496 (6.9)	58 (6.9)		211 (5.9)	51 (6.3)	
Clinical characteristics						
CES-D score, mean ± SD	12.16 ± 8.79	21.45 ± 10.27	<0.001	15.96 ± 9.94	23.86 ± 11.37	<0.001
Depression +, *n* (%)	2279 (31.9)	599 (71.1)	<0.001	1890 (53.2)	640 (78.8)	<0.001
Depression −, *n* (%)	4859 (68.1)	244 (28.9)		1661 (46.8)	172 (21.2)	

bullying +/−, the number of individuals who reported experiencing bullying within the last six months; SD, standard deviation; CES-D, Center for Epidemiological Studies Depression.

**Table 3:** Results of the logistic regression for males.

Predictor	Model 1				Model 2			
	*B*	*OR*	LCI	UCI	*B*	*OR*	LCI	UCI
Age	0.015 *	1.015	1.006	1.024	0.011 *	1.011	1.002	1.020
Education (College graduate or below)								
University graduate	0.171 *	1.186	1.058	1.330	0.151 *	1.163	1.034	1.308
Master’s degree or higher	0.002	1.002	0.849	1.182	−0.021	0.979	0.826	1.162
Marital status (Married)								
Unmarried	−0.206 *	0.814	0.716	0.926	−0.238 **	0.788	0.690	0.900
Other	0.685 **	1.984	1.360	2.895	0.663 *	1.941	1.316	2.863
Years of service	−0.009 *	0.991	0.983	0.999	−0.007	0.993	.984	1.001
Working hours	0.023 **	1.017	1.017	1.030	0.021 **	1.021	1.014	1.028
Income (Less than 3 million KRW)								
3–4 million KRW	0.140 *	1.003	1.003	1.321	0.127	1.135	0.985	1.308
Over 4 million KRW	−0.025	0.840	0.840	1.132	−0.011	0.989	0.848	1.154
Experience of bullying +					1.654 **	5.226	4.458	6.125
Nagelkerke R^2^	0.020 **				0.096 **			

*B*, estimate of the regression coefficient; *OR*, odds ratio; Experience of bullying +, the number of individuals who reported experiencing bullying within the last six months; R^2^, explanatory power; LCI, lower bound of 95% confidence interval; UCI, upper bound of 95% confidence interval. Education—College graduate or below, Marital status—Married, Income—Less than 3 million KRW, and Experience of bullying—none were set as the reference groups for the model. * *p* < 0.05, ** *p* < 0.001.

**Table 4:** Results of the logistic regression for females.

Predictor	Model 1				Model 2			
	*B*	*OR*	LCI	UCI	*B*	*OR*	LCI	UCI
Age	−0.015 *	0.985	0.974	0.996	−0.018 *	0.982	0.971	0.994
Education (College graduate or below)								
University graduate	−0.112	0.894	0.749	1.066	−0.133	0.875	0.731	1.047
Master’s degree or higher	−0.238 *	0.788	0.624	0.995	−0.259 *	0.772	0.608	0.980
Marital status (Married)								
Unmarried	−0.320 **	0.726	0.624	0.845	−0.288 **	0.749	0.642	0.875
Other	0.144	1.155	0.730	1.827	0.104	1.110	0.694	1.774
Years of service	0.012 *	1.012	1.001	1.023	0.013 *	1.013	1.002	1.024
Working hours	0.027 **	1.027	1.019	1.036	0.026 **	1.026	1.018	1.035
Income (Less than 3 million KRW)								
3–4 million KRW	0.210 *	1.234	1.046	1.456	0.233 *	1.263	1.067	1.495
Over 4 million KRW	−0.330 **	0.719	0.603	0.856	−0.297 *	0.743	0.622	0.888
Experience of bullying +					1.174 **	3.236	2.692	3.889
Nagelkerke R^2^	0.052 **				0.104 **			

*B*, estimate of the regression coefficient; *OR*, odds ratio; Experience of bullying +, the number of individuals who reported experiencing bullying within the last six months; R^2^, explanatory power; LCI, lower bound of 95% confidence interval; UCI, upper bound of 95% confidence interval. Education—College graduate or below, Marital status—Married, Income—Less than 3 million KRW, and Experience of bullying—none were set as the reference groups for the model. * *p* < 0.05, ** *p* < 0.001.

**Table 5:** Multivariate-adjusted * odds ratios (95% CI) for prevalence of depression according to experience of bullying for males and females.

	Bullying Experience −	Bullying Experience +	*p* for Interaction
Depression			
Male	1 (reference)	5.23 (4.46–6.13)	<0.001
Female	1 (reference)	3.24 (2.69–3.89)	

* Adjusted for age, education status, marital status, years of service, working hours, and income.

## Data Availability

The data necessary to interpret, replicate, and build upon the methods or findings reported in this article are available upon request from the corresponding author, S.C. The data are not publicly available due to ethical restrictions that protect patient privacy and consent.
